# Neuropsychological, immunovirological, and socioeconomic profile of transgender women living with HIV: a retrospective cross-sectional study from an Italian reference center

**DOI:** 10.3389/frph.2026.1920771

**Published:** 2026-10-14

**Authors:** Giovanni Scaglione, Federico Fama, Loredana Catinari, Claudia Conflitti, Stefania Caronni, Gaia Risso, Maria Vittoria Cossu, Marta Colaneri, Andrea Gori, Chiara Atzori

**Affiliations:** 1 Department of Infectious Disease, Luigi Sacco Hospital, Regional Center for Infectious Diseases (CEREMI), Lombardy Region, Milan, Italy; 2 Department of Biomedical and Clinical Sciences, University of Milan, Milan, Italy; 3 Centre for Studies and Research in Cognitive Neuroscience, University of Bologna, Cesena, Italy; 4 Department of Psychology, University of Bologna, Bologna, Italy; 5 National PhD Programme in One Health Approaches to Infectious Diseases and Life Science Research, Department of Public Health, Experimental and Forensic Medicine, University of Pavia, Pavia, Italy; 6 Institute of Health, School of Health Sciences, HES-SO Valais-Wallis, Sion, Switzerland; 7 The Sense Innovation & Research Center, Lausanne, Switzerland; 8 Centre for Multidisciplinary Action in Global Health (MAGH), University of Milan, Milan, Italy

**Keywords:** HIV infection, neuropsychological evaluation, people living with HIV, sex work and HIV, socioeconomic factors, transgender health

## Abstract

**Introduction:**

Transgender women experience a disproportionate burden of HIV infection and intersecting social vulnerabilities. While virological outcomes among those retained in care are increasingly documented, data integrating neuropsychological health and socioeconomic determinants, particularly in *European* settings, remain scarce. Suboptimal cognitive performance may persist despite viral suppression, potentially undermining quality of life and long-term engagement in care.

We aim to provide an integrated characterization of the immunovirological, neuropsychological, and socioeconomic profile of TW living with HIV attending a tertiary HIV reference center.

**Methods:**

We conducted a retrospective cross-sectional study at the outpatient HIV clinic of Luigi Sacco University Hospital, Milan, between 2011 and 2023. We included adult TW living with HIV who had adequate proficiency in Italian and had undergone standardized neuropsychological assessment. Clinical, immunovirological, neuropsychological, and socioeconomic data were extracted from medical records and analyzed descriptively.

**Results:**

Ninety-five participants were included, predominantly Latin American migrants (95.8%) engaged in sex work (95.6%), with high rates of regular substance use (69.5%). Most participants were virologically suppressed at the time of neuropsychological assessment (78.5%), and nearly all had CD4 counts above 200 cells/µL (97.9%). Nonetheless, performances below normative thresholds were frequently observed, particularly on tests assessing processing speed and sustained attention (45.3%) and immediate verbal memory (60.0%). Sixty percent of participants (51/85) self-reported at least one complaint across memory, attention, and mental speed domains. Among participants with below-threshold performances immunovirological characteristics appeared broadly comparable to those with preserved performances across all tests, whereas benzodiazepine use was numerically more prevalent on 6 out of 8 measures, followed by regular substance use to a lesser extent. Educational attainment showed no consistent direction across tests.

**Conclusions:**

In this clinic-based population of TW living with HIV, performances below normative thresholds across several neuropsychological measures and marked socioeconomic vulnerability were frequently observed despite generally favorable virological outcomes. These findings support the integration of neuropsychological evaluation, substance use assessment, and gender-affirming services into comprehensive, multidisciplinary HIV care models for this population.

## Introduction

People identifying as transgender women (TW) are disproportionately affected by HIV infection globally, with pooled prevalence estimates approaching 20%, and a risk of acquisition up to 66 times higher than cisgender men (1). Despite this burden, the epidemiology and clinical outcomes of HIV in TW were incorporated into European surveillance reports only in 2024, reflecting longstanding gaps in population-specific monitoring (2, 3).

In Italy, the epidemiological picture remains incompletely characterized: self-reported prevalence diverges substantially from serology-confirmed rates, and estimates derived from global meta-analyses suggest that approximately one in four TW may be living with HIV — nearly 65 times the rate observed in the general Italian population (4, 5).

Most available data on clinical and immunovirological HIV outcomes in TW derive from North American cohorts, which consistently document reduced rates of linkage to and retention in HIV care compared with cisgender individuals (6). According to the largest meta-analysis to date, suboptimal retention is associated with lower rates of viral suppression and ART adherence, reduced CD4 cell counts, and higher HIV RNA levels and co-infection rates (7). Notably, however, when linkage to care is achieved and sustained, viral suppression rates in TW appear comparable to those of cisgender men and women after adjustment for demographic factors (8).

In the Italian context, Papalini et al. confirmed several of these patterns across two teaching hospitals, yet population-level clinical data remain scarce (9). Structural factors — including migrant status, engagement in sex work, substance use, and the absence of trans-inclusive care pathways — are consistently associated with barriers to healthcare access in this population and are particularly prevalent in the Italian setting (4, 10).

HIV infection is associated with a spectrum of cognitive impairment collectively referred to as HIV-associated neurocognitive disorders (HAND), affecting an estimated 30%–50% of people living with HIV (PLWH) worldwide (11, 12). Current guidelines recommend neuropsychological (NPS) screening in PLWH, particularly in those with established risk factors including low CD4 nadir, older age, lower educational attainment, HCV co-infection, and poor ART adherence (13, 14). Although the cost-effectiveness of routine NPS screening remains debated, its clinical value is increasingly recognized in populations where multiple risk factors co-occur (15).

Transgender women living with HIV represent one such population, facing a convergence of vulnerabilities that may further amplify cognitive risk: high rates of depression, anxiety, and substance use; psychosocial stressors linked to stigma, immigration, and sex work; and widespread use of gender-affirming hormone therapies — whose benefits on mental health and gender dysphoria are well established, but whose long-term neuropsychological effects and potential interactions with key ART drugs remain poorly characterized (16–20). These intersecting factors may suggest that TW living with HIV could be at particularly high risk of cognitive vulnerability, yet they remain largely underrepresented in the neurocognitive HIV literature (7, 17, 21). To address this gap, we conducted a retrospective cross-sectional study at Luigi Sacco University Hospital in Milan, one of Italy's largest HIV reference centres.

The aim of this retrospective cross-sectional study is to provide an integrated characterization of the immunovirological, neuropsychological, and socioeconomic profile of transgender women living with HIV attending a tertiary HIV clinic in Milan, Italy, with the goal of informing more comprehensive and gender-affirming models of HIV care.

## Methods

### Study design and setting

We conducted a retrospective cross-sectional study at the outpatient HIV clinic of Luigi Sacco University Hospital, Milan, Italy, between January 2011 and December 2023.

### Study population

The study population comprised adult transgender women living with HIV and attending the outpatient HIV clinic of Luigi Sacco University Hospital. Inclusion criteria were: age ≥18 years, self-identified gender as a transgender woman, confirmed HIV diagnosis, and active clinical follow-up at our clinic. All included participants had undergone at least one standardized neuropsychological assessment during the study period. Exclusion criteria were insufficient immunovirological data or incomplete neuropsychological evaluation. NPS assessment was offered on a voluntary basis to all transgender women living with HIV attending the HIV outpatient clinic during routine follow-up visits who demonstrated adequate proficiency in Italian, as all tests were administered in Italian. Adequate Italian proficiency was assessed by clinician judgment, made by the single clinician who conducted all study visits. During the study period 127 patients in follow up were questioned and 95 accepted to undergo the NPS evaluation.

### Data collection

Clinical, laboratory, and demographic variables were extracted primarily from electronic health records (EHRs) and supplemented, when necessary, with information from paper records. Socioeconomic variables, including employment status (encompassing sex work), self-declared income, and use of tobacco, alcohol, cocaine, and cannabis use, were routinely collected using a standardized data collection form.

Pathological alcohol consumption was defined as intake exceeding two alcoholic units per day or drinking associated with social, legal, or health consequences. Regular substance use was defined as the presence of at least one of the following: pathological alcohol consumption, regular cannabis use, or cocaine use more than two times per week. This composite study-specific variable was created ad-hoc as a marker of overall substance-related vulnerability.

Syphilis-related variables were also recorded. A recent syphilis infection was defined as either a new reactive treponemal (TPHA/TPPA) or nontreponemal (RPR) test, or a reinfection evidenced by a fourfold or greater increase in RPR titer within the year preceding the neuropsychological assessment. A history of syphilis was defined as a previously documented positive treponemal test not meeting criteria for recent infection.

### Neuropsychological tests

The NPS assessment comprised four validated instruments.

1. The Trail Making Test (TMT), divided into Part A and Part B, was performed to assess psychomotor speed, attention, and cognitive flexibility (22, 23).
2. The Wechsler Adult Intelligence Scale (WAIS) IV edition, in particular the WAIS Digit-Symbol Coding (DSC) subtest, was implemented to study rapid visual processing and sustained attention (24).
3. The Rey Auditory Verbal Learning Test (RAVLT) was used to assess learning ability and immediate and delayed verbal memory (25).
4. The standard version of the Grooved Pegboard test (GP) was administered to assess fine oculomotor control, coordination, motor speed, attention, and executive functions (26). All tests were administered in Italian according to the instructions provided in the respective test manuals.

Raw scores were retained for all participants throughout 2011-2023, and no re-scoring was performed. Normative correction was applied uniformly to the full study population in 2025 using a single, consistent set of test editions and normative references. Test scores were corrected for age and years of education, and performance was assessed using published normative data. Years of education were expressed as Italian-equivalent schooling years, classified according to the International Standard Classification of Education (ISCED 2011), to allow consistent application of education-adjusted Italian normative corrections for participants educated outside Italy (27). For the TMTs, raw completion time underwent regression-based corrections for age and years of education according to Siciliano et al. (22). The obtained Equivalent Score (ES; range 0–4) was defined as below-threshold when ES ≤1. For the RAVLT, age- and education-adjusted corrections were applied to raw scores according to Carlesimo et al., likewise yielding an ES (range 0–4); below-threshold performance was defined as ES ≤1 (25). For the WAIS-IV DSC, age-adjusted corrections followed the Italian standardization, and below-threshold performance was defined as a scaled score <5 (24). For the GP, raw completion times were converted to age- and education-stratified, combined-sex Z-scores and percentiles using normative data reported by Ruff and Parker, per standard test administration and scoring procedures (28–30). Below-threshold performance was defined as a z-score ≤−1.5 relative to the normative mean. Percentile ranks were also derived and are reported descriptively in Table 3 but were not used for classification. Detailed information on cutoff values and performance classification is provided in the Online Data Supplement. Of note, these single-test classifications do not constitute a formal diagnosis of HAND according to the Frascati criteria which require evidence of functional impairment and consideration of confounding conditions in addition to cognitive impairment (12).

Prior to objective testing, a brief three-item subjective cognitive screening was administered to assess self-perceived cognitive efficiency, covering perceived changes in memory, attention and concentration, and mental speed and executive functioning. Only self-perceived “negatively impactful changes” in each of the three questions were reported as a positive answer, as opposed to “not perceived” or “perceived but not impactful” responses. Participants with suboptimal test scores were referred to the appropriate specialist for further evaluation, in line with current recommendations (14).

### Ethical approval

The study was conducted in accordance with the principles of the Declaration of Helsinki. The study protocol was approved by the Institutional Review Board of Luigi Sacco University Hospital, Milan, Italy (approval number 63037/2025, approved on 18 November 2025). Because the study involved retrospective analysis of clinical and neuropsychological data collected between 2011 and 2023, participants were contacted, where feasible, to provide written informed consent for the use of their previously collected data for research purposes. Reasonable efforts were made to contact participants, including three documented contact attempts. When participants could not be reached after these attempts, their data were included without individual consent in accordance with Article 110 of the Italian Personal Data Protection Code and the applicable safeguards and procedures approved by the competent Ethics Committee. Non-response was not interpreted as consent. All data were anonymized prior to analysis.

### Statistical analysis

Continuous variables were summarized as median and interquartile range (IQR) or mean and standard deviation (SD), as appropriate; categorical variables were reported as frequencies and percentages. Neuropsychological test performances were described according to published normative thresholds. Additional descriptive subgroup analyses were conducted for each neuropsychological test to explore the distribution of selected variables between the below-threshold and above-threshold neuropsychological performance categories, restricted to participants with complete data on all covariates considered (78/95, 82.1%). Variables of interest included age, years of education (>12 vs. others), CD4 nadir (<200 cells/µL vs. others), HIV RNA at the time of neuropsychological assessment (≥50 copies/mL vs. others), benzodiazepine use, self-declared income (>45,000€/year vs. others), and regular substance use as previously defined. Due to the exploratory and hypothesis-generating nature of the study and the limited size of the cohort, missing values were not imputed, and incomplete observations were excluded from this analysis. No inferential tests were performed because of the limited sample size.

## Results

A total of 95 participants were included. Demographic, socioeconomic, and immunovirological characteristics are summarized in Table 1 and detailed in Supplementary Table S1.

**Table 1:** Core epidemiological, HIV, and co-infection characteristics of the study population.

Variable	*N*, %
*Epidemiological data*	
Number of patients	95
Age (median [Q1, Q3])	37 [31, 43]
Country of origin	
*Brazil*	62 (65.3)
*Peru*	26 (27.4)
*Italy*	4 (4.2)
*Other Latin American country*	3 (3.2)
Years in Italy (median [Q1, Q3])	11 [4.0, 15.8]
*missing*	1 (1.05)
BMI (median [Q1, Q3])	22.8 [21.1, 25.1]
Education (years, median [Q1, Q3])	13 [8, 13]
*HIV history*	
Years since HIV Diagnosis (median [IQR])	8 [5, 14]
CD4 at NPS test (cells/µL)	
N (median [Q1, Q3])	610 [424.0, 886.5]
% (median [Q1, Q3])	28.5 [20.6, 34.5]
<200	2 (2.1)
<50	0 (0.0)
>500	59 (62.1)
HIV RNA ≥50 copies/mL at NPS test (N, %)	20 (21.5)
*Missing*	2
CD4 nadir <200 (cells/ µL) (N, %)	22 (24.7)
*missing*	6
History of opportunistic infections	42 (44.2)
ART—core drug	
*INSTI*	65/92 (70.7)
*PI*	20/92 (21.7)
*NNRTI*	7/92 (7.6)
*Missing*	3 (3.2)
*Other infections*	
History of syphilis	60/92 (65.2)
*Missing*	3 (3.2)
Recent syphilis (<12 months)	30/94 (31.9)
*Missing*	1 (1.1)
Chronic HBV infection (HBsAg+)	7/94 (7.4)
*Missing*	1 (1.1)
History of HCV infection	15 (15.8)

ART, antiretroviral therapy; BMI, body mass index, CD4, helper T cell lymphocytes, expressing CD4 co-receptor, HCV, hepatitis C virus, HBV, hepatitis B virus, HBsAg, HBV surface antigen.

The median age was 37 years (IQR 31–43) and the median years of education was 13 (IQR 8–13). The majority of participants were born in South America, predominantly Brazil (62/95, 65.3%), with a median duration of residence in Italy of 11 years (IQR 4.0–15.8). Four participants (4.2%) were born in Italy.

The median time since HIV diagnosis was 8 years (IQR 5.0–14.0). Among the 89 participants with available CD4 nadir data, 22 (24.7%) had a nadir below 200 cells/µL, of whom 7 (7.9%) had a nadir below 50 cells/µL. INSTI-based regimens were the most frequently used ART regimens (65/92, 70.7%). At the time of neuropsychological assessment, the majority of participants were virologically suppressed (73/93, 78.5%), and nearly all had CD4 counts above 200 cells/µL (93/95, 97.9%), consistent with a low risk of opportunistic infections.

Socioeconomic characteristics and trans care-related medical history are summarized in Table 2. The vast majority of participants were sex workers (SW) (87/91, 95.6%). Regarding substance use, 66 of 93 participants (71.0%) reported regular cocaine use, of whom 36 (38.7%) used the substance more than two times per week; pathological alcohol consumption was reported by 29 participants (30.5%). Regarding gender-affirming care, most participants had undergone breast augmentation (62/95, 65.3%) and reported a history of gluteal silicone injections (66/93, 71.0%). A large proportion (81/93, 87.1%) reported prior use of hormone therapy, in most cases self-prescribed.

**Table 2:** Socio-economic variables and clinical characteristics of the study population.

Variable	*N* (%)
Socio-economic variables	
Sex work	87/91 (95.6)
*Missing*	4 (4.2)
Regular substance use^Δ^	66 (69.5)
Declared annual income (*1,000€/year)	
<8	5/82 (6.1)
8–15	12/82 (14.6)
15–30	16/82 (19.5)
30–45	18/82 (22.0)
>45	31/82 (37.8)
*missing*	13 (13.7)
*Alcohol consumption*	
*No*	41 (43.2)
*Occasional consumption*	25 (26.3)
*Pathological consumption*	29 (30.5)
*Cocaine consumption*	
*Any cocaine use*	66/93 (71.0)
*missing*	2 (2.1)
>2 *times per week*	36/93 (38.7)
*missing*	2 (2.1)
*Consumption of other substances*	
Tobacco	48 (50.5)
Cannabis	37 (39.4)
*Use of psychotropic medications*	
*Benzodiazepines*	47 (49.5)
*Other*	11 (11.6)
*No*	37 (38.9)
*Gender-affirming care history*	
Mastoplasty	62 (65.3)
Gluteal silicone injections	66 (71.0)
Sex reassignment surgery	6 (6.6)
Self-prescribed hormone therapy	81 (87.1)
*Transgender care service needs*	
*Dedicated transgender care service*	40/84 (47.6)
*Mental health service*	34/84 (40.5)
*Endocrinology*	27/84 (32.1)
*Social support*	4/84 (4.8)
*Missing*	11 (11.6)

Δ Regular substance use was defined by at least one of the following: pathological alcohol consumption, regular consumption of cannabis, or cocaine >2 times per week.

Table 3 summarizes subjective cognitive complaints and objective neuropsychological test performances, corrected for age and years of education. Self-reported difficulties impacting memory, attention and concentration, and mental speed and executive functioning were common and 51 of 85 participants (60.0%) endorsed at least one of the three items.

**Table 3:** Neuropsychological test performance of the study sample.

Test	Score
Trail Making Test (TMT)	
TMT—A (median [Q1, Q3])	47 [38, 57]
*Below-threshold performance (n, %)*	10 (10.5)
TMT—B (median [Q1, Q3])	117 [93, 149.5]
*Below-threshold performance (n, %)*	12 (12.6)
TMT—B-A (median [Q1, Q3])	72 [47.5, 94.0]
*Below-threshold performance (n, %)*	15 (15.8)
Wechsler Adult Intelligence Scale (WAIS)	
WAIS—Digit symbol coding (median [Q1, Q3])	38 [31.5, 48.0]
*Below-threshold performance (n, %)*	43 (45.3)
Rey Auditory Verbal Learning Test (RAVLT)	
RAVLT—Immediate recall (median [Q1, Q3])	29.8 [24.8, 35.8]
*Below-threshold performance (n, %)*	57 (60.0)
RAVLT—Delayed recall (median [Q1, Q3])	7.2 [5.0, 8.9]
*Below-threshold performance (n, %)*	30 (31.6)
Grooved Pegboard (GP)	
Dominant hand	
GP—dominant hand mean +/ (SD)	76.4 (19.3)
*Percentile*	17 [5, 50]
*Number of drops (dominant hand)*	
0	85 (89.5)
1	4 (4.2)
2	5 (5.3)
3	1 (1.1)
*Below-threshold performance (n, %)*	21 (22.1)
Non-Dominant hand	
GP—non-dominant hand mean +/ SD	89.8 (32.3)
*Percentile*	16 [1.5, 47.5]
*Number of drops (non-dominant hand)*	
0	86 (90.5)
1	5 (5.3)
2	2 (2.1)
3	2 (2.1)
*Below-threshold performance (n, %)*	25 (26.3)
Subjective cognitive complaints	
(3-item screening)	(N, %)
0/3	34/85 (40.0)
1/3	35/85 (41.2)
2/3	12/85 (14.1)
3/3	4/85 (4.7)
*Missing*	10/95 (10.5)

TMT, Trail Making Test; WAIS, Wechsler Adult Intelligence Scale; RAVLT, Rey Auditory Verbal Learning Test; GP, grooved pegboard.

Regarding test scoring: TMT, RAVLT, and WAIS values are raw scores (with below-threshold status based on age-/education-adjusted norms as specified in Methods), Grooved Pegboard times are mean (SD), and the other score cells are median [IQR].

On the TMT-A, the median completion time was 47 s (IQR 38.0–57.0), with 10 participants (10.5%) scoring below the normative threshold. On the TMT-B, the median completion time was 117 s (IQR 93.0–149.5), with 12 participants (12.6%) scoring below the normative threshold. The median Trail B-A difference was 72 s (IQR 47.5–94.0), with 15 participants (15.8%) falling below the reference threshold.

On the WAIS-IV, the median Digit-Symbol Coding score was 38 (IQR 31.5–48.0), and below-threshold on the Digit-Symbol Coding subtest was observed in 43 participants (45.3%).

On the RAVLT, the median immediate recall score was 29.8 words (IQR 24.8–35.8), with 57 participants (60.0%) scoring below the normative threshold. The median delayed recall score was 7.2 words (IQR 5.0–8.9), with 30 participants (31.6%) scoring below the normative threshold.

Mean GP completion time was 76.4 s (SD ±19.3) for the dominant hand and 89.8 s (SD ±32.3) for the non-dominant hand, consistent with an approximately normal distribution. Performance below the normative threshold was observed in 21 participants (22.1%) for the dominant hand and in 25 participants (26.3%) for the non-dominant hand.

Descriptive subgroup analyses across all neuropsychological tests are presented in Table 4. Of note, below- and above-threshold group sizes in this table reflect the subset of patients without missing variables (78/95, 82.1%). Immunovirological characteristics were similarly distributed between below-threshold and above-threshold groups across every test. Benzodiazepine use was numerically more frequent among participants with below-threshold performance on six of eight measures (all except TMT-A and RAVLT immediate recall). Regular substance use showed the same pattern on RAVLT and WAIS Digit-Symbol Coding but reversed on TMT-B, TMT B-A, and dominant-hand Grooved Pegboard. Educational attainment showed no consistent pattern: participants with below-threshold WAIS Digit-Symbol Coding performance were substantially less likely to have more than 12 years of education (29.4% vs. 75.0%), whereas those with below-threshold RAVLT performance were numerically more likely to do so. Self-declared income likewise showed no consistent direction across tests.

**Table 4:** Descriptive analysis of demographic, clinical, and socio-behavioral factors among participants, according to their performance (below- or above-threshold) and restricted to patients without missing information (*N* = 78).

Number of patients without missing information (*N*, %)	78/95 (82.1%)	
	*Trail Making Test—A*	
	Below-threshold performance (*N* = 9)	Above-threshold performance (*N* = 69)
Age (median [Q1, Q3])	37 [36, 44]	37 [31, 43]
Education years > 12 (N, %)	4 (44.4)	39 (56.5)
CD4 nadir < 200 (cells/ µL)	3 (33.3)	18 (26.1)
HIV RNA ≥50 copies/mL at NPS test	2 (22.2)	14 (20.3)
Use of benzodiazepines	5 (55.6)	39 (56.5)
Declared annual income (> 45,000 EUR/year)	2 (22.2)	27 (39.1)
Regular substance use^a^	8 (88.9)	48 (69.6)

	*Trail Making Test—B*	
	Below-threshold performance (*N* = 9)	Above-threshold performance (*N* = 69)
Age (median [Q1, Q3])	42 [37, 44]	36 [31, 43]
Education years >12 (*N*, %)	4 (44.4)	39 (56.5)
CD4 nadir <200 (cells/ µL)	2 (22.2)	19 (27.5)
HIV RNA ≥50 copies/mL at NPS test	2 (22.2)	14 (20.3)
Use of benzodiazepines	6 (66.7)	38 (55.1)
Declared annual income (>45,000 EUR/year)	2 (22.2)	27 (39.1)
Regular substance use^a^	6 (66.7)	50 (72.5)

	*Trail Making Test—B-A*	
	Below-threshold performance (*N* = 11)	Above-threshold performance (*N* = 67)
Age (median [Q1, Q3])	42 [35.5, 45.5]	37 [31, 43]
Education years >12 (*N*, %)	6 (54.5)	37 (55.2)
CD4 nadir <200 (cells/ µL)	3 (27.3)	18 (26.9)
HIV RNA ≥50 copies/mL at NPS test	2 (18.2)	14 (20.9)
Use of benzodiazepines	8 (72.7)	36 (53.7)
Declared annual income (>45,000 EUR/year)	6 (54.5)	23 (34.3)
Regular substance use^a^	6 (54.5)	50 (74.6)

	*WAIS Digit Symbol Coding*	
	Below-threshold performance (*N* = 34)	Above-threshold performance (*N* = 44)
Age (median [Q1, Q3])	38.5 [31, 43]	36 [30.8, 44.2]
Education years >12 (*N*, %)	10 (29.4)	33 (75.0)
CD4 nadir <200 (cells/ µL)	9 (26.5)	12 (27.3)
HIV RNA ≥50 copies/mL at NPS test	5 (14.7)	11 (25.0)
Use of benzodiazepines	21 (61.8)	23 (52.3)
Declared annual income (>45,000 EUR/year)	13 (38.2)	16 (36.4)
Regular substance use^a^	25 (73.5)	31 (70.5)

	*RAVLT immediate recall*	
	Below-threshold performance (*N* = 46)	Above-threshold performance (*N* = 32)
Age (median [Q1, Q3])	36.5 [30.2, 43.0]	40 [31.8, 44.2]
Education years >12 (*N*, %)	28 (60.9)	15 (46.9)
CD4 nadir <200 (cells/ µL)	12 (26.1)	9 (28.1)
HIV RNA ≥50 copies/mL at NPS test	9 (19.6)	7 (21.9)
Use of benzodiazepines	25 (54.3)	19 (59.4)
Declared annual income (>45,000 EUR/year)	19 (41.3)	10 (31.2)
Regular substance use^a^	36 (78.3)	20 (62.5)

	*RAVLT delayed recall*	
	Below-threshold performance (*N* = 23)	Above-threshold performance (*N* = 55)
Age (median [Q1, Q3])	38 [31.0, 43.5]	36 [31, 43]
Education years >12 (*N*, %)	14 (60.9)	29 (52.7)
CD4 nadir <200 (cells/ µL)	7 (30.4)	14 (25.5)
HIV RNA ≥50 copies/mL at NPS test	3 (13.0)	13 (23.6)
Use of benzodiazepines	14 (60.9)	30 (54.5)
Declared annual income (>45,000 EUR/year)	8 (34.8)	21 (38.2)
Regular substance use^a^	22 (95.7)	34 (61.8)

	*GP—dominant hand*	
	Below-threshold performance (*N* = 17)	Above-threshold performance (*N* = 61)
Age (median [Q1, Q3])	37 [31, 42]	37 [31, 43]
Education years >12 (*N*, %)	8 (47.1)	35 (57.4)
CD4 nadir <200 (cells/ µL)	5 (29.4)	16 (26.2)
HIV RNA ≥50 copies/mL at NPS test	3 (17.6)	13 (21.3)
Use of benzodiazepines	10 (58.8)	34 (55.7)
Declared annual income (>45,000 EUR/year)	5 (29.4)	24 (39.3)
Regular substance use^a^	11 (64.7)	45 (73.8)

	*GP—non dominant hand*	
	Below-threshold performance (*N* = 19)	Above-threshold performance (*N* = 59)
Age (median [Q1, Q3])	37 [30.0, 43.5]	37 [31, 43]
Education years >12 (*N*, %)	11 (57.9)	32 (54.2)
CD4 nadir <200 (cells/ µL)	6 (31.6)	15 (25.4)
HIV RNA ≥50 copies/mL at NPS test	5 (26.3)	11 (18.6)
Use of benzodiazepines	12 (63.2)	32 (54.2)
Declared annual income (>45,000 EUR/year)	5 (26.3)	24 (40.7)
Regular substance use^a^	14 (73.7)	42 (71.2)

Below- and above-threshold group sizes in this table reflect the complete-case subset (*N* = 78) and are therefore smaller than the corresponding full-sample counts reported in Table 3.

a Regular substance use was defined by at least one of the following: pathological alcohol consumption, regular consumption of cannabis, or cocaine >2 times per week. WAIS, Wechsler Adult Intelligence Scale; RAVLT, Rey Auditory Verbal Learning Test; GP, Grooved Pegboard; CD4: helper T cell lymphocytes, expressing CD4 co-receptor.

## Discussion

This retrospective single-center cross-sectional study provides a preliminary characterization of transgender women living with HIV in Italy, integrating immunovirological, neuropsychological, and socioeconomic data. Four key findings emerge. First, most participants were sex workers (95.6%) and presented factors associated with social vulnerability, including regular substance use (69.5%) and immigrant status (95.8%). Second, HIV viral suppression (78.5%) and immune recovery (62% with CD4 counts above 500 cells/µL, 98% with CD4 counts above 200 cells/µL) were high, with most participants receiving INSTI-based ART regimens (70.7%). Third, performances below normative thresholds were frequent, particularly in tests assessing processing speed and sustained attention (45.3%) and verbal memory (60.0%).

Finally, immunovirological characteristics were similarly distributed across performance categories on every test, whereas benzodiazepine use was numerically more frequent among participants with below-threshold performance on six out of eight measures; regular substance use followed, with higher frequencies in the three tests with the highest rates of below-threshold performances, and educational attainment showed test-dependent and, in places, opposing directions.

The socioeconomic profile of the study population is consistent with prior Italian and international findings, as participants were predominantly Latin American immigrants engaged in sex work and facing structural barriers to employment, housing, and healthcare access (4, 9).

These conditions have been consistently linked in the literature to stigma, legal precariousness, delayed HIV testing, and suboptimal entry into care (5, 10). The persistent absence of gender-identity data in European surveillance systems further hinders accurate epidemiological monitoring of this population (3).

The immunovirological outcomes observed in this study are consistent with data from US-based studies demonstrating that transgender women retained in care achieve viral suppression rates comparable to those of cisgender individuals (8).

Notably, these outcomes were observed in a population characterized by high rates of substance use, sex work, and immigrant status — factors previously associated with suboptimal retention and virological outcomes in other settings (6, 7).

The proportion of participants with below-threshold performances, particularly in processing speed and sustained attention (45.3% on the WAIS Digit-Symbol Coding) and immediate verbal memory (60.0% on the RAVLT), aligns with the known vulnerability of fronto-striatal pathways in HIV-related cognitive impairment (31–33).

Below-threshold performances on individual measures were observed despite high rates of viral suppression. Chronic HIV-related neuroinflammation is one plausible contributor to this pattern, among several factors whose relative contributions cannot be disentangled in this descriptive, cross-sectional design. Notably, benzodiazepine use, reported in half of the overall sample, was the only variable examined that showed a consistent numerical direction across the majority of tests, being more frequent among participants with below-threshold performance on six of eight measures (all except TMT-A and RAVLT immediate recall). This represents a further uncontrolled pharmacological confound: benzodiazepines are well documented to acutely impair processing speed, sustained attention, and anterograde verbal memory precisely the domains assessed by the WAIS Digit-Symbol Coding and RAVLT subtests, which showed the highest rates of below-threshold performance in our study population (34). Psychosocial stress, socioeconomic vulnerability, and other unmeasured confounders discussed in the Limitations may similarly contribute, and below-threshold performance in this study should not be attributed to HIV-related mechanisms in isolation (35, 36).

Among participants with below-threshold delayed recall, regular substance use was frequent (95.7%); substance-related neurotoxicity affecting hippocampal and prefrontal integrity offers one possible explanation for this pattern (37–42). However, our substance-use measure captured chronic patterns of use rather than intoxication or withdrawal state at the time of testing, and its contribution cannot be separated from that of HIV-related mechanisms or other unmeasured confounders in this dataset. Moreover, this association was not observed uniformly: on TMT-B, TMT B-A, and the dominant-hand Grooved Pegboard, regular substance use was numerically less frequent among participants with below-threshold performance, and no consistent direction was evident across the full battery. Educational attainment showed the opposite pattern on WAIS Digit-Symbol Coding, where higher attainment was substantially more common among participants with above-threshold performance (75.0% vs. 29.4%), consistent with the concept of cognitive reserve, potentially mitigating HIV-related cognitive decline (20, 40, 43–45).

However, this pattern did not generalize: on both RAVLT measures, higher educational attainment was numerically more common among participants with below-threshold performance, and we consider a methodological explanation more plausible than a reversal of the reserve effect. Prior opportunistic infection and recent syphilis were not examined in the subgroup tables because syphilis stage was not available and cell counts would have been too small for a descriptive comparison. Gender-affirming hormone therapy was reported by 87.1% of participants, in most cases self-administered without medical supervision. Prior reviews have confirmed the psychological benefits of hormone therapy in reducing gender dysphoria, while also highlighting the potential for pharmacokinetic interactions with key ART drugs — including efavirenz, tenofovir, and ritonavir-boosted protease inhibitors — that may affect ART exposure and efficacy (20, 46).

In this study, where INSTI-based regimens predominated, the specific interactions most commonly cited above are of limited direct relevance; they should therefore remain a precautionary consideration for the minority of participants on NNRTI- or protease-inhibitor-based regimens and for any future regimen switch. Although our study was not designed to assess these interactions directly, the high prevalence of unsupervised hormone use in this population underscores the need for coordinated care pathways integrating infectious disease and endocrinology expertise to ensure both gender-affirming and antiretroviral treatment safety.

Nearly all participants were engaged in sex work. In Italy, sex work is neither legally recognized nor formally regulated, placing sex workers in a legally precarious position associated with heightened exposure to stigma, discrimination, and violence (47, 48). This, combined with over-reporting of earning may be the reasons over a third of the study population reported earnings over 45,000 Euros a year.

Despite this, irregular work limits access to healthcare services and legal protections, and may have contributed to the patterns of delayed care entry and social vulnerability observed in this and other Italian cohorts (4, 9).

A key methodological consideration concerns the use of Italian normative standards in a cohort where most participants were not born in Italy. Neuropsychological assessment in culturally heterogeneous populations is inherently complex, and no validated methodology exists for this specific context. Nevertheless, the application of Italian norms was considered appropriate for three reasons: adequate proficiency in Italian was an inclusion criterion; previous studies support the use of host-country norms when language proficiency is sufficient; and participants had resided in Italy for a median of 11 years, suggesting substantial linguistic and cultural integration (49, 50). Residual cultural and educational differences may nonetheless have influenced test performance and should be considered when interpreting these findings. Given the magnitude of the below-threshold rates, particularly the 60% rate on RAVLT immediate recall — a language- and culture-loaded task — these findings may partly reflect a mismatch between the Italian normative reference and this predominantly Brazilian and Peruvian population, rather than true impairment alone. This may also explain the divergent education pattern noted above: RAVLT and TMT were corrected for age and education, unlike WAIS Digit-Symbol Coding (age only). Education-adjusted norms raise the raw-score threshold for more educated participants; if raw RAVLT performance is depressed by the language mismatch, highly educated participants would be disproportionately pushed below threshold, while WAIS Digit-Symbol Coding would instead reflect the educational gradient directly, as observed. This is a hypothesis, not a demonstration: TMT is also education-corrected but did not show the reversal, though its subgroups (*n* = 9–11) were too small to be informative. A sensitivity analysis using Brazilian RAVLT norms was considered but not undertaken, as it would require comparing scores from the Italian-administered word list against norms derived from a distinct Portuguese-language list, with no validated equivalence between the two in the literature.

This study has several strengths. To our knowledge, it represents one of the largest clinic-based samples of TW living with HIV in Europe with integrated neuropsychological assessment. The multidimensional data collection — combining immunovirological, neuropsychological, and socioeconomic variables — provides a comprehensive characterization rarely available in this population. Neuropsychological testing was administered by a single infectious disease specialist trained by a neuropsychologist, ensuring consistency of test administration across the study period; the corresponding limitations of this single-rater approach are addressed below. Sex/gender-neutral tests or combined-sex normative references were used where available.

Numerous limitations should be acknowledged. The retrospective, single-center, cross-sectional design limits generalizability and precludes causal inference. Formal Frascati-based classification of HAND was not performed in this study; therefore, reported results should be interpreted as descriptive, test-level findings rather than as evidence of HAND prevalence in this population. As neuropsychological assessment was offered on a voluntary basis, participation bias cannot be excluded, and the analytic sample may not be fully representative of the broader clinic population. Testing was administered and scored by a single infectious disease physician trained by a neuropsychologist rather than a licensed neuropsychologist, with no formal inter-rater reliability assessment; the 12-year study period further raises the possibility of drift in administration or scoring practice over time, which this design cannot evaluate. Patients not included in our analytic sample may reflect inadequate Italian proficiency, voluntary non-participation, or incomplete evaluation, in unknown proportion; if language proficiency contributed, this may have disproportionately excluded more recently arrived, less socioeconomically integrated individuals, making our estimates a potentially conservative characterization of the broader population. Substance use was self-reported and operationalized as a composite, precluding dose- or substance-specific conclusions. No standardized depression or anxiety screening was administered despite known prevalence in this population, and HCV infection was recorded as a binary history variable without distinguishing active from cleared infection, though active replication is independently associated with cognitive impairment (51). Collectively, these uncontrolled factors limit our ability to attribute below-threshold performance specifically to HIV-related mechanisms. Detailed information on hormone therapy dosing and regimens was unavailable. Logistical changes over the study period resulted in incomplete electronic health records for some participants, requiring partial extraction from paper files and introducing potential data inconsistencies. Specific reasons for non-participation among eligible patients were not systematically recorded, precluding a formal comparison between assessed and non-assessed patients. Finally, as discussed above, residual cultural and educational differences may have influenced neuropsychological test performance despite Italian-language proficiency.

## Conclusions

This study suggests that comprehensive HIV care for transgender women should extend beyond immunovirological outcomes alone. In this clinic-based cross-sectional study, performances below normative thresholds across several neuropsychological tests and marked socioeconomic vulnerability were frequently observed despite generally favourable virological control. These findings support the integration of neuropsychological evaluation, mental health and substance use assessment, hormone therapy review, and targeted adherence support into routine HIV care for this population. Multidisciplinary care models involving infectious disease specialists, neuropsychologists, psychiatrists, and endocrinologists may help advance more equitable and person-centered transgender healthcare, as endorsed by international guidelines (52).

Cognitive stimulation interventions targeting at-risk individuals may further help strengthen cognitive reserve and mitigate HIV-related cognitive decline (53).

Prospective longitudinal studies are needed to clarify cognitive trajectories over time and the specific contributions of substance use, socioeconomic vulnerability, and hormone therapy to neuropsychological outcomes in this population. Pharmacokinetic and neuroimaging studies would help elucidate the underlying neurobiological mechanisms and inform more targeted clinical interventions.

## Data Availability

The raw data supporting the conclusions of this article will be made available by the authors, without undue reservation.
