# Supplementary material for: Neuropsychological, immunovirological, and socioeconomic profile of transgender women living with HIV: a retrospective cross-sectional study from an Italian reference center
Source: Front Reprod Health. 2026 Oct 14;8:1920771. doi: 10.3389/frph.2026.1920771 (PMC13713679; doi:10.3389/frph.2026.1920771)
Supplement: Supplementary file 1 [file Datasheet1.docx]

**Online data supplement**

**Neuropsychological, Immunovirological, and Socioeconomic Profile of Transgender Women Living with HIV: A Retrospective Cross-Sectional Study from an Italian Reference Center**

**Online Data Supplement Caption:** This file contains comprehensive supplementary material detailing neuropsychological test scoring methodologies and classification criteria, as well as the complete version of Table 1 further describing baseline epidemiological, clinical, and immunovirological characteristics of the cohort, supporting the study’s analytic framework and outcome interpretation

## **Appendix A. Neuropsychological Tests: Scoring Procedures and Classification Criteria**

### ***Appendix A1. Trail Making Test***

Raw completion times for Trail Making Test A (TMT-A), Trail Making Test B (TMT-B), and the derived difference score (TMT-B minus TMT-A) were corrected for age and years of education using the regression-based normative data provided by Siciliano et al. (2019).

Equivalent Scores were derived from standardized scores obtained after correction for age and education, yielding an ordinal five-point Equivalent Score scale. Equivalent Scores ranged from 0 to 4, where 0 indicated pathological performance, 1 borderline (suspect) performance, 2 low-normal performance, 3 normal but not optimal performance, and 4 clearly normal performance. Equivalent Scores were assigned separately for TMT-A and TMT-B.

Reduced performance was defined on the basis of Equivalent Scores, with scores equal to 0 or 1 classified as reduced performance, and scores equal to or greater than 2 considered within the normal range.

### ***Appendix A2. WAIS Symbol Digit Coding***

Raw scores obtained on each subtest were converted into scaled scores using age-specific normative tables provided in the *Wechsler Adult Intelligence Scale-Fourth Edition*, Italian Translation (Copyright © 2013). Scaled scores have a normative mean of 10 and a standard deviation of 3.

According to the guidelines provided in the WAIS-IV manual, a scaled score below 5 was considered clinically relevant. On this basis, reduced performance was defined as a scaled score (Pp) < 5. This criterion was applied to the Symbol Digit Coding subtest in order to determine the number and proportion of participants showing reduced performance.

### ***Appendix A3. Rey Auditory Verbal Learning Test (RAVLT)***

Raw scores for Immediate Recall and Delayed Recall were corrected for age and years of education using the normative tables provided by Carlesimo et al. (1996). Corrected scores were subsequently converted into Equivalent Scores (ES) according to the conversion procedures reported in the same normative study.

Equivalent Scores were expressed on a five-point ordinal scale ranging from 0 to 4, where 0 indicated pathological performance, 1 borderline (suspect) performance, 2 low-normal performance, 3 normal but not optimal performance, and 4 clearly normal performance.. Equivalent Scores were calculated separately for Immediate Recall and Delayed Recall.

For the purposes of the present study, reduced performance was defined exclusively on the basis of Equivalent Scores, with values of ES = 0 or ES = 1 classified as reduced performance, and values of ES ≥ 2 considered within the normal range. This criterion was applied separately to Immediate Recall and Delayed Recall in order to determine the number and proportion of participants showing reduced performance.

### ***Appendix A4. Grooved Pegboard Test***

Raw completion times were converted into age- and education-stratified, combined-sex standardized Z-scores and percentile ranks using the normative reference values reported by Ruff and Parker (1993). Test administration and scoring followed the standard procedure described in the test manual (Lafayette Instrument Company, 2014).

Standardized scores were used exclusively for performance classification. Reduced performance was defined as performance falling within the lower tail of the normative distribution, corresponding to a Z-score <= 1.5, indicating clinically relevant slowing. Percentile ranks corresponding to these Z-scores were also derived and are reported descriptively in Table 3 but were not used for performance classification.

**Supplementary table S1** Complete characteristics of the study population at baseline, and at neuropsychological tests screening

| **Variable** | **N, %** |
| --- | --- |
| ***Epidemiological data*** | |
| **Number of patients** | 95 |
| **Age (median [Q1, Q3])** | 37 [31, 43] |
| **Country of origin** |  |
| *Brazil* | 62 (65.3) |
| *Peru* | 26 (27.4) |
| *Italy* | 4 (4.2) |
| *Other* | 3 (3.2) |
| **Years in Italy (median [Q1, Q3])** | 11 [ 4.0, 15.8] |
| *missing* | 1 |
| **BMI (median [Q1, Q3])** | 22.8 [21.1, 25.1] |
| **Education (years, median [Q1, Q3])** | 13 [8, 13] |
| ***HIV history*** | |
| **HIV subtype** |  |
| B | 48 (72.7) |
| F1 | 6 (9.1) |
| CRF12_BF | 4 (6.1) |
| Other | 8 (12.1) |
| *missing* | 29 |
| **Years since HIV Diagnosis (median [IQR])** | 8 [5, 14] |
| **Years since HIV Diagnosis** |  |
| < 5 | 21 (22.1) |
| 6 to 10 | 38 (40.0) |
| > 10 | 36 (37.9) |
| **CD4 nadir (cells/mcL)** |  |
| N (median [Q1, Q3]) | 337 [204, 478] |
| % (median [Q1, Q3]) | 19.1 [12.7, 24.2] |
| < 200 | 22 (24.7) |
| < 50 | 7 (7.9) |
| > 500 | 16 (18.0) |
| missing | 6 |
| **CD4 at NPS test (cells/mcL)** |  |
| N (median [Q1, Q3]) | 610 [424.0, 886.5] |
| % (median [Q1, Q3]) | 28.5 [20.6, 34.5] |
| < 200 | 2 (2.1) |
| < 50 | 0 (0.0) |
| > 500 | 59 (62.1) |
| **HIV RNA (copies/mL) at NPS test** |  |
| <50 (%) | 73 (78.5) |
| 50 – 100,000 (%) | 4 (4.3) |
| 100,000 – 1,000,000 (%) | 8 (8.6) |
| > 1,000,000 (%) | 8 (8.6) |
| *missing* | 2 |
| **History of OIs** |  |
| *Pulmonary tuberculosis* | 25 (26.3) |
| *Cryptococcus infection* | 7 (7.4) |
| *Kaposi Sarcoma* | 4 (4.2) |
| *Cerebral Toxoplasmosis* | 1 (1.1) |
| *Candida Esophagitis* | 1 (1.1) |
| *Pneumocystis jirovecii Pneumonia* | 1 (1.1) |
| *Disseminated CMV infection* | 2 (2.1) |
| *Extrapulmonary tuberculosis* | 1 (1.1) |
| **TARV - core drug** |  |
| *INSTI* | 65 (70.7) |
| *PI* | 20 (21.5) |
| *NNRTI* | 7 (7.5) |
| *missing* | 3 |
| ***Co-infection history*** | |
| ***TPHA*** |  |
| Negative | 32 (34.8) |
| Positive | 60 (65.2) |
| *missing* | 3 |
| **RPR** |  |
| Negative | 64 (68.1) |
| Positive | 30 (31.9) |
| *missing* | 1 |
| **History of HBV infection (HBcAb+)** | 43 (46.2) |
| *missing* | 2 |
| **Chronic HBV infection (HBsAg+)** | 7 (7.4%) |
| *missing* | 1 |
| **History of HCV infection** | 15 (15.8) |
| **HHV8 Ab+** | 43 (53.8) |
| *missing* | 15 |

*Notes: BMI: body mass index, TCD4: helper T cell lymphocytes, expressing CD4 co-receptor, HCV: hepatitis C virus, HBV: hepatitis B virus, HBsAg: HBV surface antigen, OIs: opportunistic infections.*
